# Highly Luminescent Ternary Nanocomposite of Polyaniline, Silver Nanoparticles and Graphene Oxide Quantum Dots

**DOI:** 10.1038/s41598-019-53584-6

**Published:** 2019-11-18

**Authors:** Azza Shokry, M. M. A. Khalil, Hesham Ibrahim, Moataz Soliman, Shaker Ebrahim

**Affiliations:** 1 Department of Environmental Studies, Institute of Graduate Studies and Research, Alexandria University, P.O. Box 832 Alexandria, Egypt; 2 Department of Nanotechnology and composite materials, Institute of New Materials and Advanced Technological, City for Scientific Research and Technology Applications (SRTA- City), Alexandria, Egypt; 3 Department of Materials Science, Institute of Graduate Studies and Research, Alexandria University, P.O. Box 832 Alexandria, Egypt

**Keywords:** Chemistry, Materials science, Nanoscience and technology, Optics and photonics

## Abstract

Quantum dots (QDs) with photostability show a potential application in optical sensing and biological imaging. In this work, ternary nanocomposite (NC) of high fluorescent polyaniline (PANI)/2-acrylamido-2-methylpropanesulfonic acid (AMPSA) capped silver nanoparticles (NPs)/graphene oxide quantum dots (PANI/Ag (AMPSA)/GO QDs) have been synthesized by *in situ* chemical oxidative polymerization of aniline in the presence of Ag (AMPSA) NPs and GO QDs. Ag (AMPSA) NPs and GO QDs were prepared by AgNO3 chemical reduction and glucose carbonization methods, respectively. The prepared materials were characterized using UV-visible, Fourier transform infrared (FTIR), photoluminescence and Raman spectroscopies, X-Ray diffractometer (XRD) and high- resolution transmission electron microscopy (HRTEM). HRTEM micrographs confirmed the preparation of GO QDs with an average size of 15 nm and Ag (AMPSA) NPs with an average size of 20 nm. PANI/Ag (AMPSA)/GO QDs NC showed high and stable emission peak at 348 nm. This PANI/Ag (AMPSA)/GO QDs NC can emerge as a new class of fluorescence materials that could be suitable for practical sensing applications.

## Introduction

Polymeric nanocomposites, which incorporate advantages of both nanoparticles and polymers, have paid more attention in both academia and industry because they have outstanding mechanical and physical properties caused by the large surface area to volume ratio and high interfacial reactivity of the nanoparticles^1^.

Because of their ultrasensitivity, rapid and easy operation, much effort has been devoted to developing high fluorescent nanomaterials including gold and silver clusters, silica nanoparticles, carbon dots, graphene oxide, and graphene quantum dots^2^.

Silver nanoparticles (Ag NPs) are anti-microbial, non-toxic, chemically stable and high surface to volume ratio metal. Because of their attractive physical and chemical characteristics, Ag NPs have been extensively used in the fields of electronics, sensors and water treatment. The incorporation of the Ag NPs into the conducting polymer matrices can improve the optical, mechanical, thermal, and electrochemical properties of these polymers^3^.

Graphene oxide quantum dots (GO QDs) with a particle size ranging from 2 to 20 nm are attracting considerable attention due to their chemical inertness, low toxicity, biocompatibility, eco-friendly, high fluorescent activity, stable photoluminescence and good solubility. GO QDs can be used in sensors, optoelectronic devices and bioimaging^4^.

Polyaniline (PANI) possesses exceptional structural properties due to the inception of nitrogen heteroatom between the phenyl rings along the backbone. It has attracted much attention in numerous sensing applications such as gas detection^5^, ascorbic acid detection^6^, multi-electrode sensor arrays^7^, coatings for quartz crystal microbalance sensors^8^, chemiresistors using single-walled carbon nanotubes^9^ and as modified cladding in optical fiber sensors^10^.

Herein, the aim of this work is to synthesis Ag NPs with AMPSA as a new capping agent in aqueous medium to produce ternary PANI/Ag (AMPSA)/GO QDs NC with high emission and high stability. The produced Ag (AMPSA) NPs, GO QDs, PANI/Ag (AMPSA) NC and the ternary nanocomposite are characterized by UV-Vis and PL spectroscopies to study the optical properties, and the structural and morphological properties are investigated using FTIR, Raman, XRD and HRTEM techniques.

## Materials and Methods

### Materials

Aniline monomer (99.0%) was obtained from Research Lab (India). Ammonia solution 25% was obtained from Chem Solute (Germany). D (+) Glucose anhydrous was obtained from BDH Prolabo Chemicals. Sodium borohydride (99.0%) was received from Merck, Germany. Ammonium persulfate (APS) (98.0%) and ethanol (HPLC grade) were brought from Fisher Scientific UK. 2-acrylamido-2-methylpropanesulfonic acid (AMPSA) (97.0%) was obtained from Acros Organics (Germany). Sodium chloride (99.0%) was received from Honeywell. Silver nitrate (99.8%) was purchased from PRS Panreac, Spain. Dodecylbenzene sulfonic acid (DBSA) was purchased from El-Gomhoria Chemical Company, Egypt.

### Preparation of graphene oxide quantum dots

GO QDs were prepared by directly glucose pyrolysis. Two grams of glucose were placed into a beaker and were heated to 250 °C onto a hot plate. After 5 min, the glucose was liquated. Subsequently, the color of the liquid was changed from colorless to yellow, and then to orange through 20 min. This orange liquid was added drop by drop into 100 mL of 12.5% ammonia solution under vigorous stirring. Then the solution was heated at 70 °C for 3 hours until the odor of ammonia vanished and the pH of the solution became 7. The volume of GO QDs solution was maintained at 50 mL. The GO QDs powder was separated by heating and evaporation of the GO QDs solution at high temperature for about 2 hours.

### Preparation of AMPSA capped Ag (Ag (AMPSA)) NPs

Ag (AMPSA) NPs were synthesized by the chemical reduction of silver nitrate using sodium borohydride as a reducing agent. 1.2 mL of freshly prepared 10 mM sodium borohydride was added to 36.8 mL of deionized water in ice bath under continuous stirring. Then, 0.4 ml of 10 mM AgNO_3_ solution was added dropwise. The color of the solution gradually changed to yellow, indicating the formation of the Ag NPs. Finally, 0.3 ml of 10 mM AMPSA as a stabilizing agent was added dropwise to the mixture with a continuous stirring for 10 min. Ag (AMPSA) NPs were separated by centrifuging process (Focus serial No: 1107, Spain) at 8000 rpm for 10 min. The NPs were washed for several times using ethanol and deionized water. The collected Ag NPs were dried in a vacuum oven (GCA/precision scientific, model 10, Thelco) at 40 °C.

### Preparation of DBSA Doped PANI (PANI)

DBSA doped PANI solution was prepared by chemical oxidative polymerization of aniline. Aniline monomer (0.03 mL) was dissolved in 10 mL deionized water. Ten milliliters acidic solution of DBSA (0.3 g) and APS (0.1 g) were then slowly added through 1 h to the aniline solution with a continuous stirring at room temperature until the dark green color of colloidal solution was obtained.

### Preparation of PANI/AMPSA capped Ag (PANI/Ag (AMPSA)) NC

PANI/Ag (AMPSA) NC was prepared by *in situ* oxidative polymerization of aniline in presence of Ag (AMPSA) NPs. Aniline monomer (0.03 mL) was dissolved in 10 mL previously prepared Ag (AMPSA) NPs. Ten milliliters acidic solution of DBSA (0.3 g) and APS (0.1 g) were then slowly added to the aniline solution with a continuous stirring at room temperature until the dark green color of the colloidal solution was obtained. The prepared PANI/Ag (AMPSA) NC powder was collected by centrifuging at 7000 rpm for 8 min and washed consecutively with ethanol and deionized water. The collected NC was dried in a vacuum oven at 40 °C.

### Preparation of PANI/AMPSA capped Ag/GO QDs (PANI/Ag (AMPSA)/GO QDs) NC

PANI/Ag (AMPSA)/GO QDs NC was prepared with the same procedure PANI/Ag (AMPSA) NC was prepared as above. The ternary NC was prepared by mixing 10 mL of AMPSA capped Ag NPs and 1 mL of the previously prepared GO QDs solution under magnetic stirring for 10 min. Aniline monomer (0.03 mL) was added to the above mixture under continues stirring for 10 min. Ten milliliters of DBSA (0.3 g) and APS (0.1 g) aqueous solution was added dropwise with stirring at room temperature until the dark green colored of the nanocomposite colloidal was obtained.

### Characterization of fluorescent Ag (AMPSA) NPs, GO QDs, PANI/Ag (AMPSA) NC and PANI/Ag (AMPSA)/GO QDs NC

The absorption spectra were recorded with a UV-Visible spectrophotometer (Evolution 300, Thermo Scientific, USA). The aqueous colloidal solutions of the samples were used for obtaining the UV-Vis spectra in the range from 200 to 900 nm to determine their characteristic peaks. To examine the emission properties, a photoluminescence (PL) study of colloidal solutions was carried out. PL measurements were carried out at room temperature with fluorescence spectrophotometer (Perkin Elmer LS-55). Both the excitation and emission slits were set at 10 and 10 nm, respectively. The structural identifications and the surface modification of the samples were confirmed by the FTIR spectroscopic by using Fourier transform infrared spectrophotometer (Spectrum BX 11- LX 18–5255 Perkin Elmer). The spectra were recorded in the wavenumber range of 4000-350 cm^−1^. The crystalline structures of the prepared materials were evaluated by XRD analysis (Bruker- AXS D8 Discover) at room temperature. The Bragg angle (2 θ) has the range from 5 to 90 degrees to determine the degree of crystallinity of the prepared samples. The X-ray source was Cu target generated at 30 kV and 30 mA with scan speed 4 deg/min. Raman spectra of GO QDs and PANI/Ag (AMPSA)/GO QDs NC were measured using triple monochromatic combined with a Peltier cooled charge-coupled device detector system (Senterra Bruker). The spectra were acquired in the back-scattering geometry while the 514.5 nm line of an Ar laser was focused on the samples for excitation at a power of 2 mW, measured directly before the samples. Morphology, particle size, and selected area electron diffraction (SAED) were investigated using high resolution transmission electron microscopy (HRTEM) (JEOL, JEM-2100 LaB6). The charge of Ag (AMPSA) NPs was measured using a Zetasizer Malvern Nano-ZS. Suspension was placed in a universal folded capillary cell attached to platinum electrodes. The particle size distribution and average particle size of Ag (AMPSA) NPs were determined using particle size analyzer (Submicron Particle Size Analyzer- Beckman Coulture- N5) at 20 °C with 10.9 degree detection angle.

## Results and Discussion

### Optical properties of Ag (AMPSA) NPs, GO QDs, PANI/Ag (AMPSA) NC and PANI/Ag (AMPSA)/GO QDs NC

**Figure 1:**
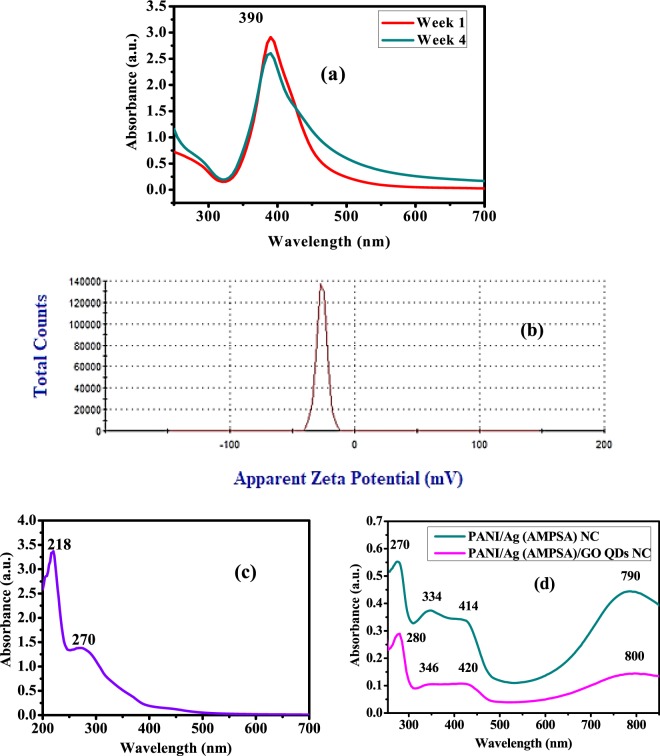
UV-Vis spectra of Ag (AMPSA) NPs at different periods (**a**), Zeta potential charts of Ag (ANPSA) NPs (**b**), UV-Vis spectra of GO QDs (**c**), PANI/Ag (AMPSA) NC and PANI/Ag (AMPSA)/GO QDs NC (**d**).

The UV-Vis spectra of Ag (AMPSA) NPs, GO QDs, PANI/Ag (AMPSA) NC, and PANI/Ag (AMPSA)/GO QDs NC are illustrated in Fig. 1. The UV–Vis absorption spectrum of Ag (AMPSA) NPs presented in Fig. 1(a) demonstrates a strong absorption peak at 390 nm, which is ascribed to the surface plasmon resonance (SPR) of Ag NPs^11,12^. The shape of the plasmon band is symmetrical and narrow confirming that Ag (AMPSA) NPs have a narrow size distribution^13^. The stability of Ag (AMPSA) NPs was determined by the intensity of this absorption peak and zeta potential. It is observed that the peak intensity of Ag (AMPSA) NPs is slightly declined after 4 weeks by ~12%. The potential value of as prepared Ag (AMPSA) NPs is −26.4 mV (Fig. 1b). The potential values greater than +25 mV or less than −25 mV typically have more stability^14^. Moreover, Mau *et al*.^15^ claimed the stability of prepared Ag NPs over one month despite decreasing their absorbance by almost 16%. The AMPSA capping agent acts as a stabilizer and preserves the Ag NPs from the photodegradation.

The UV–Vis absorption spectrum of GO QDs suspended solution exhibits two absorption peaks centered at 218 and 270 nm as shown in Fig. 1(c). These peaks are attributed to π electron transition in the C=O and C=C groups containing GO QDs. More specific, the 218 nm high peak has resulted from π → π* transition of C=C and the 270 nm small peak is due to n → π* transition of the C=O bond^4,16^.

The UV-Vis spectra of PANI/Ag (AMPSA) NC and PANI/Ag (AMPSA)/GO QDs NC are presented and compared in Fig. 1(d). The characteristic bands of doped PANI at about 270, 334, 414 and 790 nm are observed in the PANI/Ag (AMPSA) NC spectrum. Absorption peak located around 270 nm is recognized via the chain of the aromatic nuclei and corresponds to the π-π* transitions^17^. The small peak around 334 nm can be also attributed to the π−π* transition of benzenoid rings^18,19^. The small shoulder around 414 nm is due to the polaronic transition (polaron-π*) of protonated polyaniline^18^^.^ In addition, the broad peak located around 790 nm is attributed to polaron band transition (π-polaron) on PANI backbone^18^. It is noted that these characteristic peaks appear in PANI/Ag (AMPSA)/GO QDs NC with a small red shift in these peaks position after the addition of GO QDs. Moreover, the peak of Ag NPs (390 nm) is overlapped with the characteristic peaks of PANI (270–280) in PANI/Ag (AMPSA) and PANI/Ag (AMPSA)/GO QDs NC. The absorption peak of GO QDs (270 nm) is also overlapped with the peak of PANI (420) in PANI/Ag (AMPSA)/GO QDs NC.

**Figure 2:**
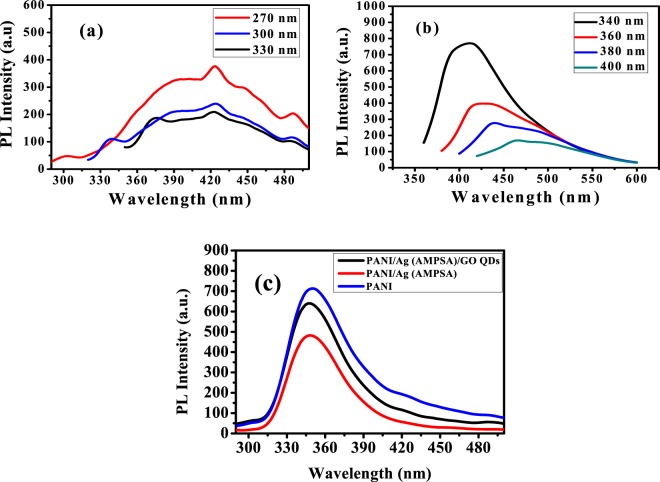
PL spectra of Ag (AMPSA) NPs (**a**), GO QDs (**b**) at different λ _ex_, and PANI, PANI/Ag (AMPSA) NC and PANI/Ag (AMPSA)/GO QDs NC (**c**) at λ_ex_ = 270 nm.

To examine the emission properties, PL spectra of a fixed volume (100 μL of stock solutions in 3 mL deionized water) of the prepared Ag (AMPSA) NPs, GO QDs, PANI/Ag (AMPSA) NC and PANI/Ag (AMPSA)/GO QDs NC are carried out at room temperature (Fig. 2). The emission spectra can be used to explain the recombination process of photogenerated electrons and holes by the fluorescence intensity. The high emission intensity corresponds to recombination of photogenerated charge carriers with a short lifetime. The separation of the photogenerated carriers, electrons (e^−^) and holes (h^+^), is high due to a longer lifetime, that leads to diminishing the intensity in the PL spectra^19^.

The PL spectra of Ag (AMPSA) NPs aqueous solutions at different excitation wavelengths are shown in Fig. 2(a). Ag NPs with size larger than 2 nm exhibit a localized surface plasmon resonance and are normally non-luminescent^20^. In this work, it is noted that fluorescence spectrum with a broad peak is recorded for Ag (AMPSA) NPs (20 nm). The HRTEM imaging and SAED techniques were explored to further probe the fine structures of obtained luminescent Ag NPs. The luminescent Ag (AMPSA) NPs have polycrystalline structures, as will be shown later from HRTEM images and contain small domains. These small size domains result in discrete energy states that lead to the luminescence^20,21^. In contrast, the average domain sizes of non-luminescent large Ag (AMPSA) NPs are greater than 2 nm.

By excitation of a sample of Ag (AMPSA) NPs with several wavelengths varied from 270 nm to 330 nm, broad emission bands from about 350 to 490 nm and a small sharp peak at 426 nm are shown. This sharp peak is attributed to the SPR of Ag NPs^20,22^. The peak positions of the PL emission of Ag (AMPSA) NPs are fixed as the excitation wavelength changes. Additionally, the intensities of the PL peaks decrease with progressively longer excitation wavelengths^23^. However, this excitation wavelength-independent PL behavior of Ag (AMPSA) NPs is in contrast with the other published data of Ag NPs, in which their PL emission peak positions are shifted and depended on the excitation wavelengths^20,22^. The maximum emission intensity of Ag (AMPSA) is found at **λ**_**ex**_ of 270 nm.

Figure 2(b) shows the PL spectra of GO QDs at different excitation wavelengths. Broad PL bandwidth can appear when the sample is excited under different wavelengths. The PL mechanism of GO QDs is a combination of PL components from four types of electron transitions, σ*-n and π*-n transitions dominated by the functional groups, π*- π transitions of the aromatic cores and π*-midgap states-π transitions^24^. The strongest signal at 413 nm is observed with an excitation wavelength of 340 nm where this shorter wavelength with higher photon energy is more effective for photon excitation. The PL peaks are shifted from 413 to 466 nm and their intensities are decreased as excitation wavelengths exceeded from 340 to 400 nm. This excitation-dependent PL behavior was extensively reported in fluorescent carbon-based nanomaterials^25,26^ and is caused by the electronic conjugate structures, free zigzag sites and the wide distributions of differently sized dots^26,27^.

PL spectra of PANI, PANI/Ag (AMPSA) NC and PANI/Ag (AMPSA)/GO QDs NC aqueous solutions at excitation wavelength of 270 nm are demonstrated in Fig. 2(c). PANI aqueous solution exhibits high PL. The origin of PL in PANI is due to the delocalized π -conjugated electrons and π*- π transition of the benzenoid unit of polyaniline^28^.

The presence of Ag (AMPSA) NPs during the PANI polymerization reduces the PL intensity of pristine PANI due to the destructive spectral overlapping^29^. However, the PL intensity of the PANI/Ag (AMPSA)/GO QDs NC is improved, since the radiative recombination rate is increased by the coupling of the surface plasmon in the Ag NPs and GO QDs^24,30,31^ as shown in Fig. 2(c). Matching plasmon resonance of Ag NPs to the emission spectrum of GO QDs is essential for achieving efficient enhancement of PL^24^. This complex nanostructures composed of Ag NPs concentrates the photon energy in a small region, which significantly enhances the local electromagnetic field. The area affected by the enhanced electromagnetic field, called a “hot spot”, contributes to amplify the weak emission signal^32^.

The most obvious mechanism for the PL enhancement of PANI/Ag (AMPSA)/GO QDs NC due to GO QDs adsorption onto PANI/Ag (AMPSA) NC is based on the electrostatic interaction and the van der Waals forces. The large amount of negatively charged groups such as carboxyl, aldehyde and hydroxyl on the GO QDs and the positively charged amine groups of PANI/Ag (AMPSA) NC allows relatively strong electrostatic interaction. Such electrostatic effects can be considered as the main reason for the interaction between GO QDs and PANI/Ag (AMPSA) NC. Besides, functional groups like −OH and −NH_2_ could work as the donor or acceptor of hydrogen bonds. This leads to the aggregation, which passivates the surface defect states of GO QDs and the PL intensity of the NC is enhanced^33,34^. It can be concluded that the PANI/Ag (AMPSA)/GO QDs NC has high PL intensity thanks to the synergistic effect of the constituents of the ternary composite involved PANI, GO QDs and Ag (AMPSA) NPs.

The room-temperature PL quantum yield (QY) of PANI/Ag (AMPSA)/GO QDs NC was determined by comparing the integrated emissions of the NC samples in aqueous solution with those of standard fluorescent “L-tryptophan” with an identical optical density. The QY of PANI/Ag (AMPSA)/GO QDs NC is 0.138 ≈ 14%. This value is similar to the QY of the standard L-tryptophan that reported in the literature^35^. For the QY estimation, the Eq. (1) is used^36,37^. Where F and F_std_ are the PL areas in the sample and the standard amino acid (L-tryptophan), respectively; A and A_Std_ are the absorbance of the NC and L- tryptophan and; n and n_std_ are the refraction index of the NC and L- tryptophan. The QY of the standard L-tryptophane is 0.14^35^.

1 $${\varnothing }_{{\boldsymbol{F}}}={\varnothing }_{{\boldsymbol{F}}({\boldsymbol{Std}})}\ast \frac{{\boldsymbol{F}}\,\ast \,{{\boldsymbol{A}}}_{{\boldsymbol{Std}}}\,\ast \,{{\boldsymbol{n}}}^{2}}{{{\boldsymbol{F}}}_{{\boldsymbol{Std}}}\,\ast \,{\boldsymbol{A}}\,\ast \,{{{\boldsymbol{n}}}^{2}}_{{\boldsymbol{Std}}}}$$

The refractive indices of PANI/Ag (AMPSA)/GO QDs NC and L-tryptophan were measured using Abbe refractometer.

### Stability of PANI/Ag (AMPSA)/GO QDs NC

Stability of the fluorescent materials is a vital parameter for the application of PANI/Ag (AMPSA)/GO QDs NC as a fluorescent probe sensor. To ensure the stability of PANI/Ag (AMPSA)/GO QDs, the effect of ionic strength on the fluorescence of PANI/Ag (AMPSA)/GO QDs NC is examined in the presence of various concentrations of NaCl. Also, the fluorescence spectra of PANI/Ag (AMPSA)/GO QDs NC are measured after stored for different periods at room temp (~30 °C).

#### Effect of ionic strength

**Figure 3:**
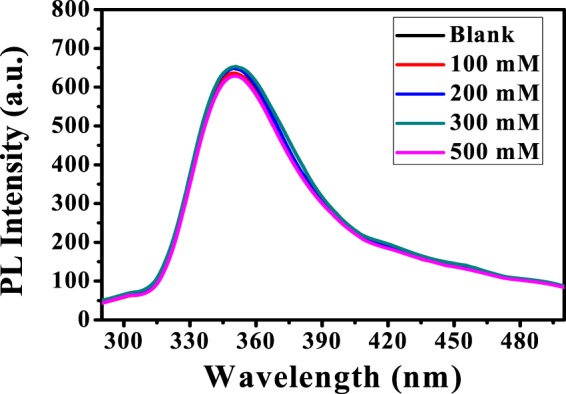
PL spectra of PANI/Ag (AMPSA)/GO QDs NC vs. NaCl at pH 6, λ_ex_ = 270 nm.

The influence of ionic strength on fluorescence intensity of synthesized PANI/Ag (AMPSA)/GO QDs is studied using various concentrations of NaCl from 100 to 500 mM as presented in Fig. 3. It is observed that ionic strength has no significant effect on fluorescence intensity which is evidence that there is no interaction between the nanocomposite and NaCl. Results are demonstrated that PANI/Ag (AMPSA)/GO QDs NC have a stabilized fluorescence intensity under different ionic strength and it is a good candidate as fluorescent sensor applications.

#### Effect of time

**Figure 4:**
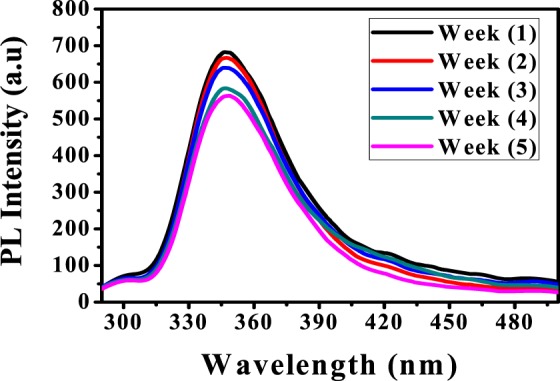
PL spectra of PANI/Ag (AMPSA)/GO QDs NC vs. time at pH 6, λ_ex_ = 270 nm.

To investigate the fluorescence stability of the prepared PANI/Ag (AMPSA)/GO QDs with time, the PL intensity of PANI/Ag (AMPSA)/GO QDs solution is weakly measured for 5 weeks. Results are revealed that PANI/Ag (AMPSA)/GO QDs solution exhibit high resistance to photobleaching and the fluorescence intensity is slightly dropped by 7.3% and 16.3% after three and five weeks, respectively as depicted in Fig. 4. The high stability of the PL of PANI/Ag (AMPSA)/GO QDs may be due to the presence of AMPSA capping agent or the high stable PL of GO QDs itself. Also, the PANI/Ag (AMPSA)/GO QDs NC solution remains homogeneous and dispersed without any aggregation or color change.

### Structural properties of Ag (AMPSA) NPs, GO QDs, PANI/Ag (AMPSA) NC and PANI/Ag (AMPSA)/GO QDs NC

**Figure 5:**
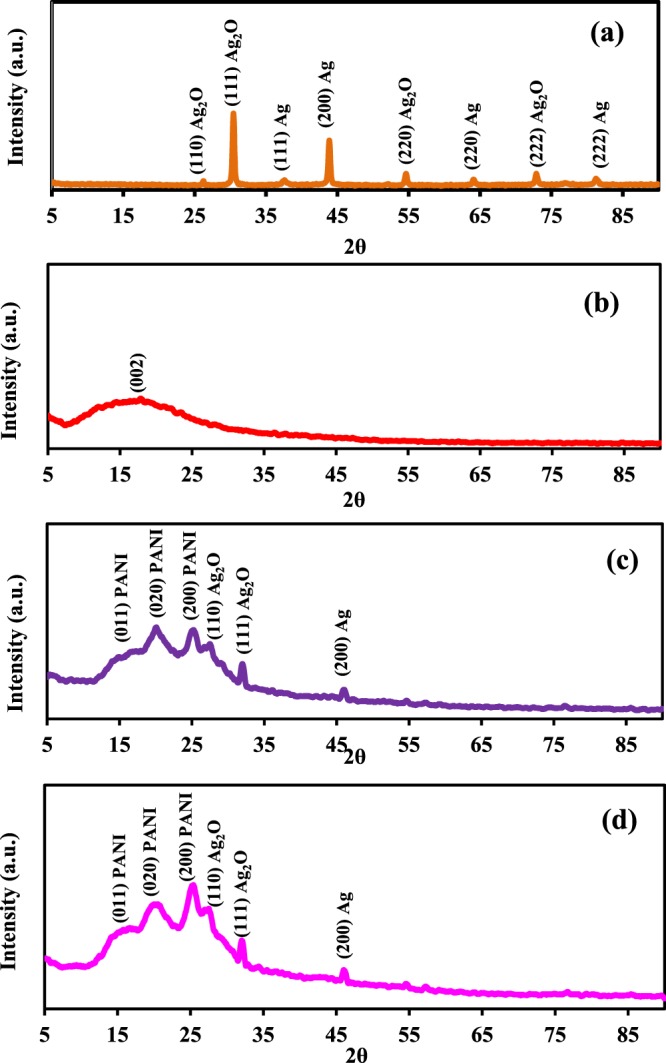
XRD patterns of Ag (AMPSA) NPs (**a**), GO QDs (**b**), PANI/Ag (AMPSA) NC (**c**) and PANI/Ag (AMPSA)/GO QDs NC (**d**).

X-ray diffraction is a powerful technique used to identify the phases (crystalline or amorphous) presented in the material. The crystallinity of Ag (AMPSA) NPs, GO QDs, PANI/Ag (AMPSA) NC and PANI/Ag (AMPSA)/GO QDs NC was investigated using XRD as shown in Fig. 5.

The XRD pattern of Ag (AMPSA) NPs (Fig. 5a) confirms the formation of Ag NPs. At 2θ values of 37.63°, 43.92°, 64.83° and 81.20° several Bragg reflection peaks are indexed to (111), (200), (220), (222), respectively and is matched with the standard spectrum of Ag NPs (Joint Committee on Powder Diffraction Standards (JCPDS) No. 00-004-0783)^18,38,39^. Also, small four peaks are observed at 2θ values of 26.16°, 30.46°, 54.60° and 72.76° indexed to (110), (111), (220) and (222), respectively and correspond to presence of cubic configuration of Ag_2_O NPs^40^.

The XRD pattern of GO QDs illustrated in Fig. 5(b) shows a characteristic broad diffraction peak (002) centered at 2θ = 17.62°. This broad peak also indicates that the prepared GO QDs have a small particle size^16,41^. This is also mainly due to the presence of oxygenated groups, which increased the d-space between graphene sheets^16,25,42–44^.

XRD patterns of PANI/Ag (AMPSA) and PANI/Ag (AMPSA)/GO QDs nanocomposites shown in Fig. 5(c,d, respectively) depict the dominants characteristic peaks of PANI in the form of emeraldine salt. There are two broad peaks at 17.30°, 19.72° and a small sharper peak at 25.22° corresponding to the (011), (020) and (200) lattice planes of PANI chains, respectively. The first peak at 17.30° is attributed to parallel repeat units of PANI. The other two peaks at 19.72° and 25.22° are attributed to the periodicity parallel and perpendicular to the polymer chains of PANI, as well as to a periodicity caused by H-bonding between PANI chains^40,45^. There are also some small peaks characteristics for both Ag_2_O and Ag NPs with an obvious decrease in their peaks intensity in comparison with those of pristine Ag (AMPSA) NPs. This is maybe due to the amorphous polymer coating and shielding the Ag (AMPSA) NPs^46^. Another interesting aspect is that the peak of Ag NPs at 43.92° in the Ag (AMPSA) NPs XRD pattern shifts to a higher 2θ in the nanocomposites. According to Blanton and Majumdar^47^, the 2θ peak can shift due to the oxygen functional groups on the Ag (AMPSA) NPs surface that facilitates the interaction between PANI and Ag (AMPSA) NPs. Another reason for the peak shift is the slight stretching of the unit cell of Ag NPs due to the adsorption of PANI molecular chains on the surface of the Ag (AMPSA) NPs^48^. For the XRD pattern of PANI/Ag (AMPSA)/GO QDs NC, the diffraction peak of GO QDs at 17.62° is overlapped with the peak of PANI at 17.30° (Fig. 5d).

**Figure 6:**
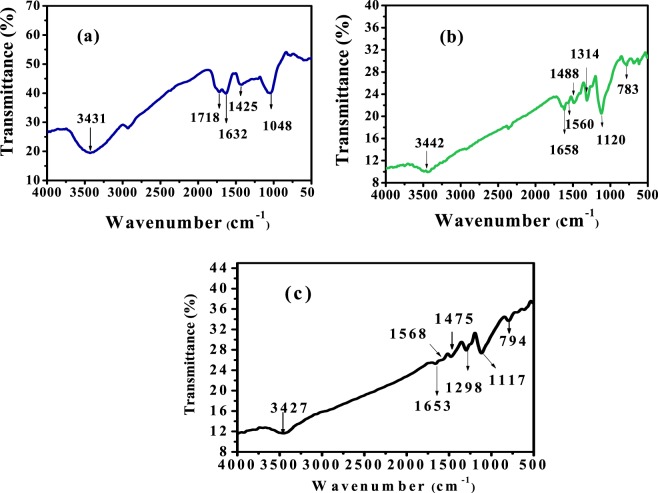
FTIR spectra of GO QDs (**a**), PANI/Ag (AMPSA) NC (**b**) and PANI/Ag (AMPSA)/GO QDs NC (**c**).

FTIR technique was used to determine the functional groups of GO QDs, PANI/Ag (AMPSA) NC and PANI/Ag (AMPSA)/GO QDs NC as illustrated in Fig. 6. The FTIR spectrum of GO QDs (Fig. 6a) shows a band at about 1632 cm^−1^ corresponding to the aromatic C=C stretching vibration. At the same time, the characteristic peaks at about 3431, 1718, 1425, and 1048 cm^−1^ reveal the presence of –OH, C=O, C–OH, and C–O groups, respectively. These oxygen functional groups are characteristic of the oxidized forms of graphene^4,25,26^.

The infrared spectrum of PANI/Ag (AMPSA) NC presented in Fig. 6(b) demonstrates the presence of bands of PANI. The small broad band at 3442 cm^−1^ represents the N-H stretching mode^45,49^. The two peaks appear around 1560 cm^−1^ and 1488 cm^−1^ are assigned to C=C stretching vibration of the quinoid ring and C=C stretching vibration of the benzenoid ring, respectively^49–51^. The band at 1314 cm^−1^ is assigned to the C–N single bond stretching in benzenoid ring^50,51^. The peak at 1120 cm^−1^ corresponds to the vibration of (−NH^+^=) group resulted in the DBSA doping process of polyaniline^45,49,51^, while the peak at 783 cm^−1^ is associated with C–H out-of-plane bending vibrations of the para-substituted benzene ring^50–52^. The spectrum of PANI/Ag (AMPSA) NC also shows the C=O stretching peak of AMPSA monomer at 1658 cm^−1 ^^53^.

The FTIR spectrum of PANI/Ag (AMPSA)/GO QDs NC (Fig. 6c) confirms the presence of PANI in the nanocomposite and there are no obvious characteristic bands of GO QDs in the PANI/Ag (AMPSA)/GO QDs. This is maybe due to those vibrational bands of PANI shield or interference with the bands of GO QDs. It is notable that the spectrum of PANI/Ag (AMPSA)/GO QDs NC is similar to the spectrum of PANI/Ag (AMPSA) NC. These results confirm the successful preparation of GO QDs, PANI/Ag (AMPSA) NC and PANI/Ag (AMPSA)/GO QDs NC. The degree of oxidation can be predicted depending on the relative intensities of FTIR absorption peaks of benzenoid and quinoid stretching vibrations. These peaks have a ratio of about 1:1 in the doped PANI of the PANI/Ag (AMPSA) and PANI/Ag (AMPSA)/GO QDs NC as shown in Fig. 6(b,c, respectively). This shows that the doping level of the PANI is 50%^54^.

**Figure 7:**
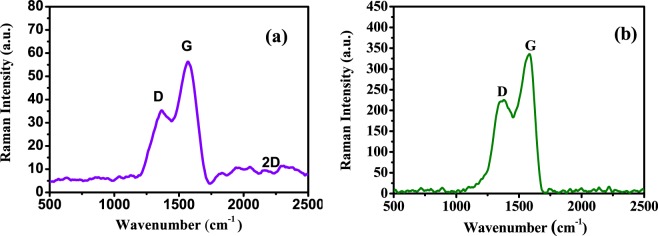
Raman spectra of GO QDs (**a**) and PANI/Ag (AMPSA)/GO QDs NC (**b**).

Raman spectroscopy is used to analyze information related to the electronic and structural properties of GO QDs and PANI/Ag (AMPSA)/GO QDs NC. It is a powerful tool for the characterization of carbonaceous materials. Figure 7(a) depicts the Raman spectrum of GO QDs and the major Raman features of GO QDs are the D band at around 1324 cm^− 1^, the high G band at 1589 cm^− 1^ and the small broad 2D band at around 2236 cm^−1^. The D band represents the defect in graphitic structure^55–57^ and the G band represents the symmetric vibration of carbon atoms in graphite structure^57^. The intensity ratio of D band and G band (*I*_D_/*I*_G_) represents the defect density in carbon structure^58^. It is found that *I*_D_/*I*_G_ for the prepared GO QDs is only around 0.6 and is similar to that of high quality few-layer graphene nanoribbons, which indicates the high quality of the prepared GO QDs^41^. In the Raman spectrum of PANI/Ag (AMPSA)/GO QDs NC shown in Fig. 7(b), the intensity ratio of the two bands (*I*_D_/*I*_G_), at 1348 cm^−1^ and 1585 cm ^−1^ is higher than GO QDs (about 0.7). This suggests that the defect in the PANI/Ag (AMPSA)/GO QDs NC is raised. It is noticed that D band is slightly shifted to higher wavenumber in PANI/Ag (AMPSA)/GO QDs NC and this is attributed to the interaction between GO QDs and PANI and Ag NPs in PANI/Ag (AMPSA)/GO QDs NC. However, other vibrational peaks of PANI are not appeared because of overlapping with GO peaks^59^.

### Morphological properties of Ag (AMPSA) NPs, GO QDs, PANI/Ag (AMPSA) NC and PANI/Ag (AMPSA)/GO QDs NC

**Figure 8:**
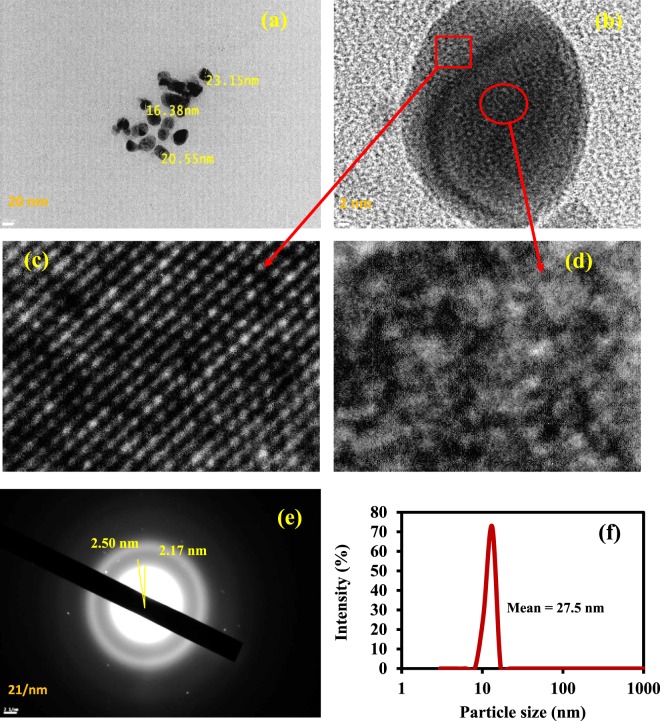
HRTEM images of aggregated Ag (AMPSA) NPs (**a**), high magnified Ag (AMPSA) NPs (**b**), luminescent Ag (AMPSA) NPs (**c**), non-luminescent Ag (AMPSA) NPs (**d**) SAED image of Ag (AMPSA) NPs (**e**) and the particle size distribution of Ag (AMPSA) NPs (**f**).

**Figure 9:**
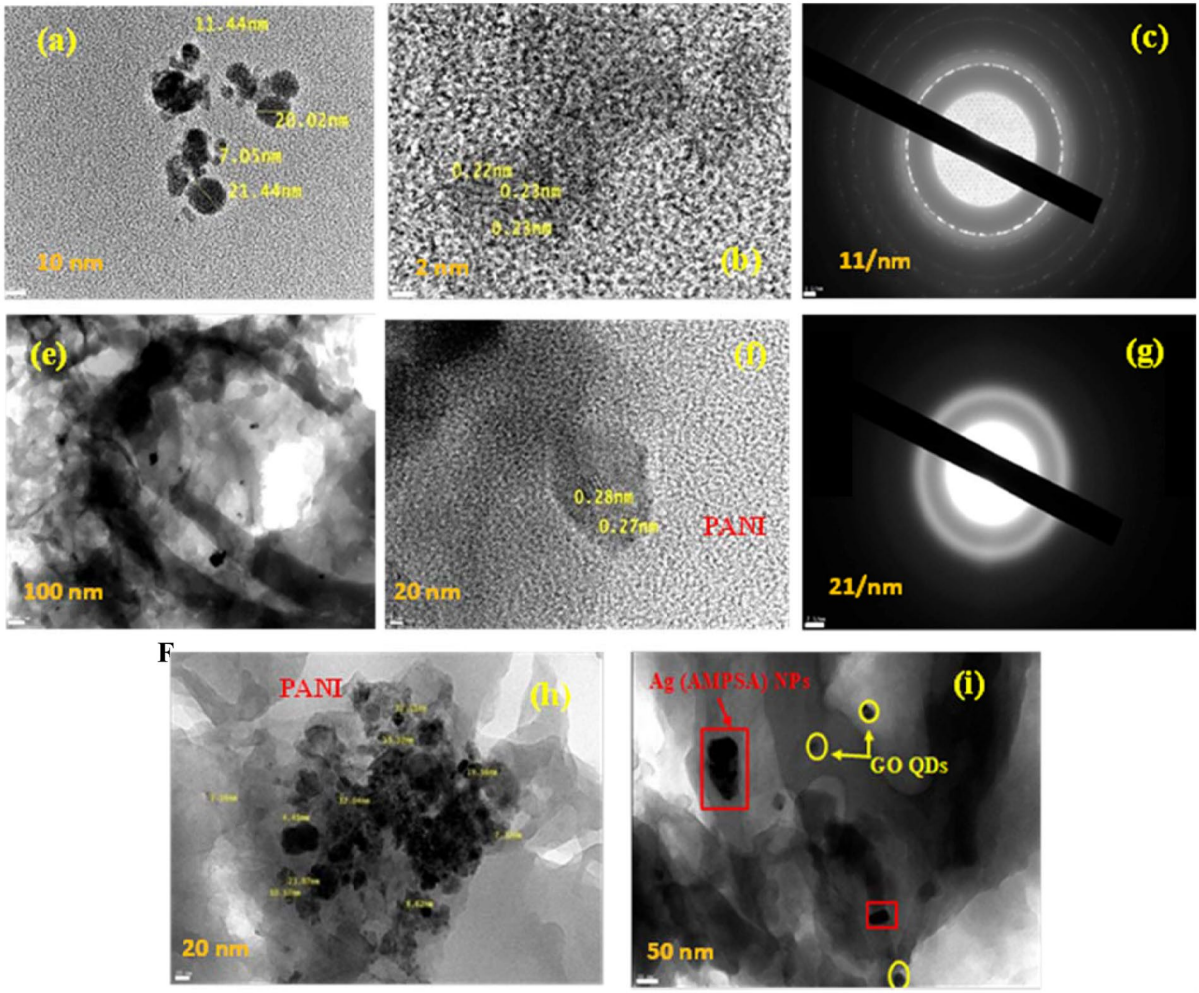
HRTEM images of GO QDs (**a,b**), SAED of GO QDs (**c**), PANI/Ag (AMPSA) NC (**e,f**), SAED of PANI/Ag (AMPSA) NC (**g**) and PANI/Ag (AMPSA)/GO QDs NC (**h,i**).

Morphological features of the as-synthesized Ag (AMPSA) NPs, GO QDs, PANI/Ag (AMPSA) NC and PANI/Ag (AMPSA)/GO QDs NC are verified by HRTEM as shown in Figs. (8) and (9). HRTEM image of Ag (AMPSA) NPs shown in Fig. 8(a) indicates that there are aggregations of Ag NPs, although the presence of AMPSA as a capping agent. The shapes of the nanoparticles are nearly oval with an average size of 20 nm. Figure 8(b) demonstrates polycrystalline domains of Ag (AMPSA) NPs that contain luminescent crystals (Fig. 8c) and non- luminescent Ag (AMPSA) NPs as illustrated in Fig. 8d. Also, the SAED of Ag (AMPSA) NPs presented in Fig. 8(e) confirms that Ag (AMPSA) NPs are polycrystalline with a d-spacing of ~0.33 nm. To confirm the size distribution of Ag (AMPSA) NPs, the particle size analyzer was used. It is clear that the size distribution (Fig. 8f) is a narrow and the average particle size (estimated by fitting the distribution spectrum using the Gaussian distribution function) is ~27.5 nm.

Morphological features of the as-synthesized GO QDs, PANI/Ag (AMPSA) NC and PANI/Ag (AMPSA)/GO QDs NC are verified by HRTEM as shown in Fig. 9. HRTEM image of GO QDs (Fig. 9a) displays spherical GO nanoparticles with an average size of 15 nm and the obvious crystal lattice space presents a high crystallinity of GO QDs with d-space of 0.23 nm as depicted in Fig. 9(b). The observed SAED of GO QDs shown in Fig. 9(c) consists of concentric rings that show the polycrystalline structure of the GO QDs^60^.

HRTEM images of PANI/Ag (AMPSA) NC presented in Fig. 9(e,f) confirm the existence of Ag (AMPSA) NPs in the PANI matrix and the well- resolved lattice space with a d-spacing of ~0.27 nm of Ag (AMPSA) NPs clarifies their crystallinity. Moreover, the SAED of PANI/Ag (AMPSA) nanocomposite is displayed in Fig. 9(g). The hollow circles in the pattern confirm the amorphous structure of PANI. HRTEM images of PANI/Ag (AMPSA)/GO QDs NC at different magnifications shown in Fig. 9(h,i) reveal that it is composed of sheets of PANI as a matrix including Ag (AMPSA) NPs (red rectangles) and GO QDs (yellow spheres), respectively.

## Conclusions

The successful synthesis and modification of new Ag (AMPSA) NPs and GO QDs with PANI was confirmed by UV-Vis, PL, Raman and HRTEM. The average particle size of Ag (AMPSA) NPs was 20 nm with an oval structure. The addition of PANI to GO QDs and Ag (AMPSA) NPs tuned the PL emission where PANI/Ag (AMPSA)/GO QDs aqueous solution exhibited a sharper emission peak at 348 nm when excited with a wavelength of 270 nm. Furthermore, the PL intensity of this novel NC showed high stability towards the ionic strength and time. It was concluded that PANI/Ag (AMPSA)/GO QDs NC would provide great potential for a wide range of applications such as sensors and energy technology.
